# Spotlight on MuSK positive myasthenia gravis: clinical characteristics, treatment and outcomes

**DOI:** 10.1186/s12883-022-02593-6

**Published:** 2022-03-04

**Authors:** Qi Huang, Feng Li, Song Zhao

**Affiliations:** Department of Thoracic Surgery, The First Affiliated Hospital of Zhengzhou University, 1 Jianshe East Road, Zhengzhou, 450003 Henan China

**Keywords:** Myasthenia gravis, Autoantibody, Muscle specific receptors tyrosine kinase, Clinical features, Long-term follow up

## Abstract

**Background:**

To investigate the clinical characteristics, treatments and outcomes of patients with myasthenia gravis with antibodies to muscle-specific tyrosine kinase (MuSK-MG).

**Methods:**

We retrospectively reviewed the cases of 21 patients with confirmed MuSK-MG between January 2012 and January 2020 in our centre. Detailed clinical data and long-term follow-up information were summarized.

**Results:**

Females (17/21, 81%) predominated among these MuSK-MG patients, and the mean age of onset in this group was 51.86 ± 16.16 years. MuSK-MG patients were divided into three subgroups according to the symptoms of muscle weakness at onset: ocular myasthenia gravis (OMG, 47.6%), bulbar myasthenia gravis (BMG, 42.9%), and generalized myasthenia gravis (GMG, 9.5%). The mean progression time from symptom onset to other muscle group involvement in OMG patients was 4.38 ± 2.54 months. Pyridostigmine bromide was adopted in 81.0% of patients, and 90.5% of patients received corticosteroids. Compared to usage in hospitals, the median daily dose of corticosteroids decreased significantly at the last follow-up. A total of 85.7% of patients received a long-term follow-up, with an average time of 1202.17 ± 976.73 days. At the end of the follow-up period, 4.8% of patients had achieved complete stable remission, 42.9% of patients had minimal manifestations, 19.0% had improved, the condition of 4.8% of patients remained unchanged, and 9.5% of patients died.

**Conclusion:**

Female patients were more prevalent in this study, and MuSK-MG patients rapidly progressed to a generalized state. Although approximately 50% of MuSK-MG patients can achieve a favourable outcome with conventional immunosuppressants, complete stable remission is rare, and approximately 15% respond poorly. More effective medications should be explored in these patients.

**Supplementary Information:**

The online version contains supplementary material available at 10.1186/s12883-022-02593-6.

## Background

Myasthenia gravis (MG) is an autoimmune disease that affects the neuromuscular junction and usually leads to skeletal muscle weakness and fatigability [1, 2]. Patients with MG who have no detectable circulating antibodies (Abs) to acetylcholine receptor (AChR) are frequently defined as having seronegative MG (SNMG) [2]. In 2001, a novel serum antibody against muscle-specific tyrosine kinase (MuSK) was revealed in SNMG patients and was found in 70% of AChR-Ab-seronegative MG patients [3]. As a subgroup of MG, MuSK-Ab-positive patients often suffer more severe bulbar muscle weakness and present various clinical characteristics. Although previous reports have shown that the symptoms of MuSK-MG patients may be associated with region and race [4, 5], studies about the relevant clinical features and treatment outcomes in Asians are rare [6]. Hence, the specific disease course, especially in response to standard treatment and the prognosis of MuSK-MG patients, still needs further observation and investigation. We retrospectively analysed the clinical characteristics, treatment and prognosis of MuSK-MG patients in our medical centre in China, aiming to improve clinicians’ further understanding of the disease.

## Methods

### Study design and data collection

This was a retrospective single-centre study. This study was approved (2020-KY-231) by the ethics committee of the First Affiliated Hospital of Zhengzhou University, and written informed consent for the use of clinical data was waived by the ethics committee. The clinical data of patients who were diagnosed with MG at the First Affiliated Hospital of Zhengzhou University from January 2012 to January 2020 were retrospectively reviewed and documented in a well-constructed MG database. The baseline characteristics were collected from the hospital information system. Autoantibody tests were double checked with the data from the Department of Neuroimmunology in the Henan Institute of Medical and Pharmaceutical Sciences. The inclusion criteria were a confirmed diagnosis of MG and seropositivity for MuSK Ab. Patients were regularly followed up at the outpatient department, and those who stopped visiting us were called for the last follow-up in January 2021. The clinical, diagnostic, therapeutic, and prognosis data of these patients, including sex, the age of onset, initial symptoms, disease progression, clinical classification, Myasthenia Gravis Foundation of America (MGFA) clinical class, disease severity, pharmacological findings, electrophysiological findings, serological findings, thymus examination result, comorbidities, therapeutic options, and prognosis, were analysed.

### Patients and diagnostic criteria

This study comprised 21 patients with confirmed MuSK-MG, 4 males and 17 females. All patients were diagnosed with MG by typical myasthenic symptoms with diurnal variation and at least one of the following results: positive pharmacologic response to oral pyridostigmine or corticosteroids; positive repetitive nerve stimulation (RNS) test; and seropositive for AChR Ab or MuSK Ab or low-density lipoprotein receptor-related protein (LRP4) Ab. For each participant, the MGFA classification was used to evaluate the clinical status and disease severity at the onset of myasthenic symptoms and at each follow-up. The MGFA Postintervention Status was used to assess the clinical state after treatment: complete stable remission (CSR), pharmacologic remission (PR), minimal manifestation (MM), and change in status [7].

### Detection of AChR-ab and MuSK-ab

AChR-Ab levels were tested using a standard AChR-Ab ELISA Kit (RSR, UK) following the manufacturer’s instructions, with a cut-off value of 0.45 nM. All sera were measured by a radioimmunoassay (RIA) for MuSK antibody detection. The MuSK-Ab RIA Kit (RSR, UK) was used following the manufacturer’s instructions. A cut-off value of 0.05 nM was used to score MuSK-positive samples [8].

### Statistical analysis

IBM SPSS statistics 21 (IBM Corp., Armonk, NY, USA) was used for statistical analysis. Continuous variables are presented as the means (SD), and categorical variables are summarized as numbers and frequencies. Normally distributed continuous variable analysis was performed by Student’s *t* test. The Mann-Whitney test was used for nonnormally distributed data analysis, and the chi-square test was used for categorical variables data analysis. A *P* value less than 0.05 was considered statistically significant.

## Results

Detailed clinical data of the 21 MuSK-MG patients are listed in Table 1.

**Table 1:** Clinical features of MuSK MG patients

Characteristic	Males (***n*** = 4)	Females (***n*** = 17)	Total (***n*** = 21)
Mean age in years (SD)	66.25 ± 8.62	49.24 ± 16.68	52.48 ± 16.75
Mean age at onset in years (SD)	66.00 ± 8.41	48.53 ± 15.86	51.86 ± 16.16
First symptom			
Ocular	2/4	8/17	10/21
Bulbar	1/4	7/17	8/21
Neck	0/21	1/21	1/21
Limb	1/4	1/17	2/21
Clinical subtypes at onset			
Ocular	2/4	8/17	10/21
Generalized	2/4	9/17	11/21
MGFA status at onset			
I	2/4	8/17	10/21
II	1/4	8/17	9/21
III	1/4	1/17	2/21
IV	0/4	0/17	0/21
V	0/4	0/17	0/21
AChR positive	0/4	1/17	1/21
RNS abnormalities	2/4	14/17	16/21
Thymoma present	0/4	0/17	0/21
Thymectomy	0/4	0/17	0/21
Myasthenic crises	1/4	5/17	6/21
Neostigmine test	3/4	15/17	18/21
Medications at early follow up			
Pyridostigmine bromide	1/4	5/17	6/21
Corticosteroids	2/4	9/17	11/21
Tacrolimus	2/4	0/17	2/21
Azathioprine	0/4	2/17	2/21

A total of 21 patients were included in this study. Females predominated in this group (17/21, 81%). The mean age at onset was 51.86 ± 16.16 (66.00 ± 8.41 years for male patients and 48.53 ± 15.86 years for female patients). The median follow-up time in the whole group was 1134.05 ± 928.86 (range 230–3440) days, and 3 patients refused to provide any information at the last follow-up.

### Initial symptoms of MuSK-MG

At onset, 10 patients had ocular muscle weakness (ptosis, 6/21 (28.6%); double vision, 5/21 (23.8%)), 8 had bulbar weakness (dysphagia, 4/21 (19.0%); dysphonia, 4/21 (19.0%)), 1 had respiratory muscle weakness, 1 had neck muscle weakness and 2 had limb weakness. According to the symptoms of muscle weakness at onset, MuSK-MG patients were divided into the following groups: ocular myasthenia gravis (OMG, predominant manifestations are ptosis and diplopia), 10 patients (47.6%); bulbar myasthenia gravis (BMG, predominant manifestations are impaired speech, difficulty swallowing or chewing and shortness of breath), 9 patients (42.9%), and generalized myasthenia gravis (GMG, predominant manifestation is limb weakness), 2 patients (9.5%).

### Testing

Neostigmine testing was performed in 90.5% of patients and was positive in 57.9% of those tested, occasionally with cramps and fasciculations. Electrodiagnostic testing was performed by repetitive nerve stimulation (RNS) in 76.2% of patients, with a positive rate of 37.5%. Abnormal performance in RNS testing was frequently shown in facial and proximal upper extremity muscles. All 21 patients underwent mediastinum CT scans as a routine examination. AChR-Ab and MuSK-Ab expression coexisted in patient 1 (a 50-year-old female who presented with ptosis at onset and had minimal manifestations at the last follow-up after a series of treatments). No thymomas were detected, and the anti-titin Ab test showed a consistent result (negative in 100%).

### Serial studies of clinical status

The details of treatment and the patients’ physical conditions throughout the course of the disease are reported in Table 2. Disease severity grade was measured according to the MGFA classification during the disease course (at onset, in the maximally deteriorated state, after or during treatment). Table 2 provides serial drug dosages and MGFA scores during the long-term follow-up. We recorded the time from the first symptom to the involvement of other muscle groups and the time from onset to myasthenic crisis. The ten OMG patients all (100%) had generalized weakness progression, and the mean progression time from the onset of symptoms to other muscle group involvement was 4.38 ± 2.54 months. Six patients (4/6 OMG patients, 2/6 BMG patients) suffered from an MG crisis and were hospitalized, and the median time from onset to myasthenic crisis was 7.76 ± 4.48 months. As the disease progressed, the severity grades of OMG patients (grade I at onset) increased in a short period; 7/10 (70%) patients had extraocular muscles involved (MGFA grade IIb), and 3/10 (30%) patients experienced difficulty swallowing or breathing (MGFA grade IIIb).

**Table 2:** Detailed treatment and MGFA evaluation during disease course of MuSK MG patients

N	Gender	Age at onset (years)	Duration (days)	MGFA classification	Treatment		
					PYR	CS	T acro
**(1) OMG patients**							
1	female	48	0	I	N	N	N
			30	IIb, GE^b^	N	N	N
			82	NA	Y	Y	N
			87	V, MC	Y(240 mg)	Y(45 mg)	N
			360	IIa	Y(120 mg)	Y(10 mg)	N
2	male	71	0	I	N	N	N
			150	IIIb, GE	NA	N	N
			260	NA	Y	Y	N
			350	V, MC	Y(360 mg)	Y(60 mg)	N
			380	died			
3	female	50	0	I	N	N	N
			76	IIb, GE	N	Y	N
			95	I	N	Y(80 mg)	N
			300	0^*^	N	N	N
			1020	0	N	N	N
4	female	37	0	I	N	N	N
			25	I	Y(180 mg)	N	N
			74	I	Y(270 mg)	Y(60 mg)	N
			180	I^a^	Y(360 mg)	Y(40 mg)	N
			210	IIb, GE	Y(180 mg)	Y(30 mg)	Y
			306	0	Y(90 mg)	Y(25 mg)	Y
5	female	31	0	I	N	N	N
			180	IIb, GE	N	N	N
			385	IIb	N	N	N
			393	NA	N	Y	N
			565	IIb	Y(180 mg)	Y(60 mg)	N
			1105	0*	N	N	N
			2903	0	N	N	N
6	female	40	0	I	N	N	N
			120	IIb, GE	N	N	N
			502	NA	Y	Y	N
			521	IIb	Y(240 mg)	Y(52 mg)	Y
			1855	IIb	N	N	N
7	female	49	0	I	N	N	N
			30	NA	Y	Y	N
			270	IIIb, GE	Y	Y	N
			279	V, MC	Y(240 mg)	Y(20 mg)	N
			510	IIIb	Y(480 mg)	Y(48 mg)	N
			860	0	N	Y(15 mg)	N
8	female	59	0	I	N	N	N
			60	IIa, GE	N	N	N
			146	NA	Y	Y	N
			1610	I	Y(180 mg)	N	Y
			2240	I	N	N	Y
			2810	IIb	N	Y(56 mg)	Y
			3440	IIIb	N	Y(60 mg)	N
			3762	IIa	N	Y(15 mg)	N
9	male	57	0	I	N	N	N
			157	IIa, GE	N	Y	Y
			226	I	N	Y(48 mg)	Y
			325	I	Y(30 mg)	Y(30 mg)	Y
10	female	57	0	I	N	N	N
			60	IIIb, GE	N	Y	N
			259	V, MC^a^	N	Y(45 mg)	N
			630	died			
**(2) BMG patients**							
11	female	50	0	IIb	N	N	N
			270	IIb	Y(90 mg)	N	N
			420	0^*^	N	N	N
12	female^^^	66	0	IIb	N	N	N
			25	IIb	Y	Y	N
			40	IIb	Y(180 mg)	Y(10 mg)	N
13	female	36	0	IIIb	N	N	N
			20	NA	Y	Y	N
			50	V, MC	Y(360 mg)	Y(40 mg)	N
			750	IIIb	Y(180 mg)	Y(50 mg)	N
			1255	0	N	Y(20 mg)	Y
14	female	63	0	IIb	N	N	N
			371	V,MC^#^	Y	Y	N
			380	IIIb	Y(180 mg)	Y(50 mg)	N
			1320	0	Y(60 mg)	Y(15 mg)	N
15	male	75	0	IIIb	N	N	N
			192	NA	Y	Y	N
			198	IIIb	Y(180 mg)	Y(60 mg)	N
			543	0	Y(180 mg)	Y(15 mg)	N
16	female	68	0	IIb	N	N	N
			1065	NA	Y	Y	Y
			1490	IIb	Y(180 mg)	Y(50 mg)	Y
			2525	0	Y(60 mg)	Y(10 mg)	Y
17	female^^^	16	0	IIb	N	N	N
			216	NA	Y	Y	N
			223	IIb	Y(180 mg)	Y(50 mg)	N
			230	IIb	Y(180 mg)	Y(50 mg)	N
18	female	30	0	IIb	N	N	N
			18	NA	N	Y	N
			36	IIb	N	Y(50 mg)	N
			840	IIa	N	Y(50 mg)	N
			910	IIa	N	N	N
19	female	78	0	IIb	N	N	N
			366	NA	Y	Y	N
			370	IIb	Y(180 mg)	Y(20 mg)	N
			1170	IIb	Y(180 mg)	Y(20 mg)	N
**(3) GMG patients**							
20	male^^^	61	0	IIa	NA	N	N
21	female	47	0	IIa	N	N	N
			206	NA	Y	Y	N
			210	IIb	Y(240 mg)	Y(60 mg)	N
			1095	IIa	Y(60 mg)	Y(20 mg)	N

*PYR* pyridostigmine bromide, *CS* corticosteroids, *Tacro* tacrolimus, *NA* not available, *GE* symptoms processed to generalized myasthenia gravis, *MC* MG crisis

^*^ Chinese herbal medicine; ^#^ IVIg (intravenous immunoglobulin); ^a^AZA (azathioprine, 100 mg); ^b^plasm exchange 5 times; ^^^patients lost at the last follow up

### Treatment

**Fig. 1:**
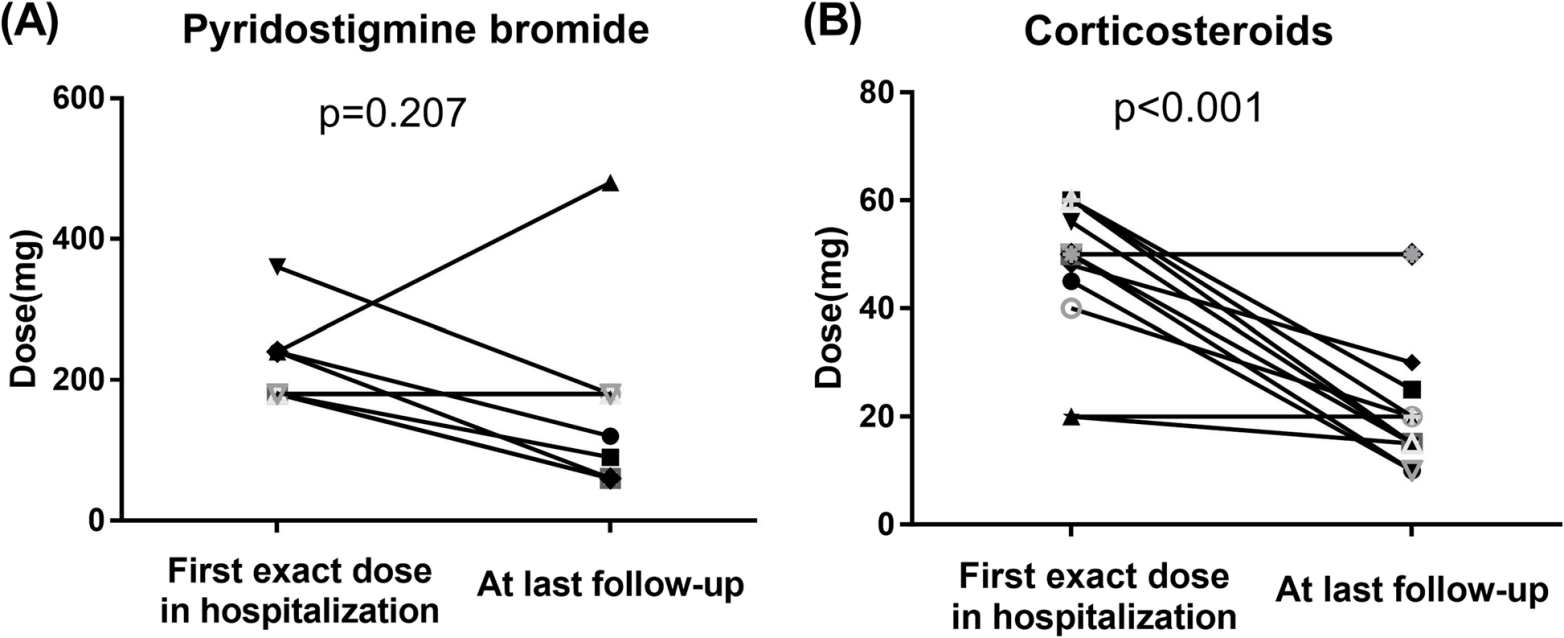
Comparison of daily medications of MuSK MG patients between the hospitalization period and at last follow-up. Daily dose of pyridostigmine bromide (**A**), corticosteroids (**B**) between the hospitalization period and at last follow-up

The detailed treatment at each time point during the disease course is shown in Table 2. According to the guidelines, AChE-I (acetylcholinesterase inhibitor) treatment using pyridostigmine bromide was adopted in 17 patients (81.0%) as a symptomatic medication. To present the regulation of drug dosage on MuSK-MG patients, we recorded the exact dose of pyridostigmine bromide and corticosteroids during the follow-up (Fig. 1). The average dose in early follow-up was 225 mg (15 patients received ≥180 mg during the severe condition). Compared to BMG patients, the median daily dose of pyridostigmine bromide (258.8 ± 138.8 vs. 191.3 ± 75.1 mg, *P* = 0.09) did not change significantly in OMG patients. Nineteen patients (90.5%) received corticosteroids, and the median peak dose of OMG patients was 55.8 ± 10.7 compared with BMG patients (42.5 ± 17.5, *P* = 0.16). Compared to hospital usage, the median daily dose of corticosteroids decreased significantly (Fig. 1B, 46.8[20–60] vs. 22.7[10–50] mg, *P* < 0.001) at the last follow-up; however, the median daily dose of pyridostigmine bromide did not change significantly (Fig. 1A, 216[180–360] vs. 159[60–480] mg, *P* = 0.207). The combination of pyridostigmine bromide, corticosteroids and immunosuppressants was adopted as the main therapeutic pattern, and 16/21 (76.2%) patients received combination therapy after the onset of symptoms. Six patients received calcineurin inhibitors (tacrolimus) combined with pyridostigmine bromide and corticosteroids. Two patients received antimetabolites (azathioprine); however, this application did not have a satisfactory therapeutic effect, and both patients experienced disease progression. One patient underwent plasma exchange therapy (five times) and experienced a myasthenic crisis in 1 month (MGFA V). One patient received IVIg when she suffered a myasthenic crisis. Interestingly, at follow-up, three patients adopted Chinese herbal medicine instead of pyridostigmine bromide or corticosteroids. With the maintenance of herbal medicine, the symptoms of the patients were stable for a long time without progression.

### Prognosis

Eighteen MuSK-MG (85.7%) patients had a long-term follow-up, with an average time of 1202.17 ± 976.73 (range 306–3762) days (Table 3). The mean follow-up period of OMG patients was slightly longer (1240.10 ± 1215.31) than that of the BMG patients (1163.29 ± 693.86, *P* = 0.88). At the end of the follow-up period, long-term outcomes were generally favourable (Table 3): 1 (4.8%) patient had achieved complete stable remission (CSR), 9 (42.9%) patients had minimal manifestations (MM), 4 (19.0%) had improved, the disease status of 1 (4.8%) patient remained unchanged, and 2 (9.5%) patients died during the follow-up period. Females predominated among CSR or MM patients (female:male = 8:2). Thirteen (61.9%) patients received long-term therapy with monotherapy or drug combinations (pyridostigmine bromide, corticosteroids and tacrolimus). We noticed that at the last follow-up, four patients continued tacrolimus as a maintenance treatment complemented by pyridostigmine bromide or corticosteroids, and all of them (100%) achieved an MGFA-PIS of MMs.

**Table 3:** Relevant features of MuSK MG patients at last follow-up

Characteristic	Males (***n*** = 4)	Females (***n*** = 17)	Total (***n*** = 21)
Thymectomy	0/4	0/17	0/21
Treatment outcome			
CSR/PR/MM	0/0/2	1/0/7	1/0/9
Improved	0	4	4
Unchanged	0	1	1
Worse	0	0	0
Died	1	1	2
Follow up time in days (SD)	416.00 ± 113.37	1359.40 ± 998.83	1202.17 ± 976.73
Medications at last follow up			
Pyridostigmine bromide	2/4	8/17	10/21
Corticosteroids	2/4	11/17	13/21
Tacrolimus	1/4	3/17	4/21
Azathioprine	0/4	2/17	2/21

Two patients (2/21, 9.5%) in this study died during the follow-up period. A 72-year-old male patient received treatment with pyridostigmine bromide (360 mg/day) and corticosteroids (60 mg/day), but he eventually died from myasthenia gravis with MGFA V. Another patient was a 58-year-old woman, dying from disease generalization in spite of the corticosteroid and azathioprine treatment.

## Discussion

In this study, we systematically reviewed 21 patients diagnosed with MuSK-MG in our centre, and the detailed clinical characteristics, including therapy and disease progression time, were recorded. We evaluated the treatment response and the severity of disease on the basis of the MGFA grade. Considering the significance of the long-term study of MuSK-MG, the present study provides a better understanding of MuSK-MG disease and its clinical course. Previous reports of the age of the onset of MuSK-MG have varied considerably between ethnic groups. The onset age of MuSK-MG in Caucasians reported in European and American countries is mostly younger than 40 years [4], whereas in Asians, the age of onset is generally after 40 years [6, 9, 10]. The mean age of onset of MuSK-MG in the present study was 51.86 ± 16.16 years, which was consistent with previous studies in Asia [10]. In contrast to the varying age of onset, MuSK-MG patients showed a similar female predominance (M:F = 4:17). Previous studies indicate that the functions of oestrogen in enhancing B-cell production and promoting antibody secretion may be related to the sex ratio [11].

The initial muscle symptoms and the progression of MuSK-MG patients have been reported differently. A US study found that only 36% of patients with MuSK-MG started with ocular muscle symptoms and developed frequent crises early in the course of the disease, all of which progressed to a generalized form within 5 years [4]. Other studies revealed that MuSK-MG rarely starts as simple extraocular muscle weakness, with symptoms concentrated in the bulbar muscles [12]. In the present study, 48% of patients with MuSK-MG started with extraocular muscle weakness, and 100% of patients progressed to weakness in other muscle groups in 4.38 ± 2.54 months, further confirming the risk of early progression to the generalized condition of MuSK-MG [13]. In addition, 6/21 patients suffered from myasthenic crises, and as previously reported, the crisis rates ranged from 25 to 48% in America [14]. Notably, 67% (4/6) of these patients had mild onset symptoms, with an MGFA class of I before deterioration.

In recent years, several centres have reported the clinical characteristics of MuSK patients (see Additional file 1). These studies have shown that long-term AChE-I treatment in patients with MuSK-MG is a first-line treatment for improving symptoms, with an effectiveness rate of 16–75% in patients with Caucasian MuSK-MG; however, there are significant individual differences in these effects [15]. Studies suggested that the improvement in the symptoms and prognoses depends on early and aggressive treatment and long-term maintenance and that the combination of drugs is effective [16, 17]. In this study, 16 patients were treated with corticosteroids in combination with AChE-I in the early stages, and 56.3% of patients achieved long-term symptomatic stability or remission. Similar results were published by Zhang et al., but Lavrnic et al. reported that MuSK-positive patients in their centre were all treated with corticosteroid drugs with a response rate of 47% [6, 18]. Furthermore, our study indicates that, low doses of pyridostigmine bromide and corticosteroids seem to be helpful for symptomatic maintenance in MuSK-MG patients.

Previous study reminded the application of rituximab in the treatment for MG showed a probability of a favourable outcome, especially in refractory patients [19]. However, in consideration of the price and health insurance in the local area, no patients in this study received rituximab. In addition, tacrolimus was administered in 6 patients, and 4/6 patients were receiving the drug as a maintenance therapy at the last follow-up. Although a CT scan of the mediastinum was performed as a common practice, no patient received thymectomy in our study due to the fact that previous multicentre cohort indicated that thymectomy was not associated with additional clinical improvement in MuSK patients [20].

Most (85.7%) patients in this study had a long-term follow-up (range 306–3762 days), and the status at the end of the observation period showed a good result compared with previous studies [4, 18]. Two patients were treated with IVIg or plasma exchange while experiencing an MG crisis and received satisfactory clinical results, suggesting the importance of timely and feasible treatment in an urgent condition. Jeffrey et al. confirmed the clinical improvement with IVIg and plasma exchange therapy and recommended that the effect of plasma exchange therapy was more rapid and dramatic for acute exacerbations than that of IVIg therapy [4]. However, two patients died during the study period, reminding physicians that older age, combined underlying diseases and physical condition may be factors in their poor prognosis, and classic therapy only has an effect on prolonging the prognosis to some extent. The 9.5% mortality reminds physicians that MuSK-MG is still intractable. Patients with refractory symptoms and unsatisfactory responses to classic treatment need considerable care, and further exploration of effective therapies is urgent.

## Conclusions

In summary, our review of the cases of 21 MuSK-MG patients illustrated the clinical presentations and long-term outcomes of the treatment strategies used in these patients. Female patients were more prevalent, and the involvement of extraocular muscle occurred as a primary initial episode. However, these patients rapidly progressed to a generalized state. The use of combination treatment might be helpful for symptom improvement and clinical outcome. However, some patients with MuSK Abs responded poorly to standard care. Therefore, early introduction of more effective treatments should be considered once a diagnosis is confirmed.

## Supplementary Information

**Additional file 1.** [21, 22].

## Data Availability

The datasets used and/or analyzed during the current study are available from the corresponding author on reasonable request.

## Abbreviations

MG: Myasthenia gravis
AChR: Acetylcholine receptor
MuSK: Muscle-specific tyrosine kinase
MGFA: Myasthenia Gravis Foundation of America
CSR: Complete Stable Remission
PR: Pharmacologic Remission
MM: Minimal Manifestation
OMG: Ocular myasthenia gravis
BMG: Bulbar myasthenia gravis
GMG: Generalized myasthenia gravis
AChE-I: Acetylcholinesterase inhibitor
