# Supplementary material for: Spotlight on MuSK positive myasthenia gravis: clinical characteristics, treatment and outcomes
Source: BMC Neurol. 2022 Mar 4;22:73. doi: 10.1186/s12883-022-02593-6 (PMC8895578; doi:10.1186/s12883-022-02593-6)
Supplement: Supplementary file 1 — Additional file 1. [21, 22]. [file 12883_2022_2593_MOESM1_ESM.docx]

**Additional file 1** Summary of relevant studies that focused on the clinical features and treatment of MuSKAb-positive patients

| **References** | **Country** | **Date** | **Number** | **Pyridostigmine therapy** | **CS therapy** | **Therapy response evaluation** | **Thymectomy** | **Follow-up** | **Status at the end of**  **observation period** |
| --- | --- | --- | --- | --- | --- | --- | --- | --- | --- |
| Sanders, D B et al.[21] | USA | 2003 | 12 | 75% | ＞41.7% | ND | 58.3% | ND | ND |
| Evoli, Amelia et al.[22] | Italy | 2003 | 37 | most | 81.1% | MGFA classification | 40.5% | At least 1 year | 3 CSR;4 PR;4 MM;  21 improved;  3 unchanged;2 died |
| Lavrnic, D et al.[18] | Serbia | 2005 | 17 | most | 100% | ND | 52.9% | Yes | 11.8%CSR;23.5%PR;  29.4%improved;  23.6%unchanged;  11.7%died |
| Lee, Jee-Young et al.[9] | Korea | 2006 | 4 | ND | 100% | MGFA classification | 25% | ND | 100%improved |
| Ohta, K et al.[6] | Japan | 2007 | 23 | ND | 52.2% | MGFA classification | 13.0% | ND | 3 PR;6 improved;  3 unchanged |
| Guptill, Jeffrey T et al.[4] | USA | 2010 | 110 | 98% | 92% | MGFA classification | 36% | 11 years for the Rome patient; 5.3 years for the Duke patients | 7.3%CSR;6.4%PR;40.4%MM,  37.6%improved;  7.3%unchanged;0.9%Worse |
| Li, Mingqiang et al.[8] | China | 2018 | 2 | 100% | 100% | ND | 50% | ND | 50%MM;50% improved |
| Zhang, Zunwei et al.[10] | China | 2020 | 14 | 57.1% | 100% | MGFA classification | ND | 11.8±11.0 months | 7.7%CSR;23.1%PR; 53.8%improved;15.4%unchanged |

CS, corticosteroids; ND, not described; CSR, complete stable remission; PR, pharmacologic remission; MM, minimal manifestations
